# Mediation of the association of smoking and microvascular complications by glycemic control in type 1 diabetes

**DOI:** 10.1371/journal.pone.0210367

**Published:** 2019-01-07

**Authors:** Barbara H. Braffett, Madeline Murguia Rice, Heather A. Young, John M. Lachin

**Affiliations:** 1 Department of Epidemiology & Biostatistics, George Washington University, Washington, D. C., United States of America; 2 The Biostatistics Center, George Washington University, Rockville, Maryland, United States of America; Universidad Miguel Hernandez de Elche, SPAIN

## Abstract

Studies have demonstrated the adverse effects of smoking on the risk of microvascular complications; however, few have also examined the potential mediating effects of glycemic control. Using data from the Diabetes Control and Complications Trial (DCCT 1983–1993), we describe the acute and long-term risks of smoking on glycemic control and microvascular complications in a well-characterized cohort of participants with type 1 diabetes. The DCCT recorded self-reported smoking behaviors, glycemic exposure based on HbA1c, and complications status. Generalized linear mixed models were used to assess whether time-dependent measurements of smoking predict HbA1c levels. Cox proportional hazard models were used to assess time-dependent smoking exposures as predictors of retinopathy and nephropathy. During a mean of 6.5 years of follow-up, current smokers had consistently higher HbA1c values and were at a higher risk of retinopathy and nephropathy compared with former and never smokers. These risk differences were attenuated after adjusting for HbA1c suggesting that the negative association of smoking on glycemic control is partially responsible for the adverse association of smoking on the risk of complications in type 1 diabetes. These findings support the potential for a beneficial effect of smoking cessation on complications in type 1 diabetes.

## Introduction

The Diabetes Control and Complications Trial (DCCT) and the United Kingdom Prospective Diabetes Study have demonstrated that improved metabolic control can prevent or delay long-term morbidity and mortality in individuals with diabetes [1, 2]. The prevalence of diabetes continues to increase worldwide [3, 4] and despite advances in treatment, it is estimated that approximately one third of all individuals with diabetes are affected by diabetic retinopathy and nephropathy [5–7]. Minimizing the risk of long-term complications and premature mortality has become a global priority.

Cigarette smoking is associated with a heightened risk of morbidity and mortality in individuals with diabetes [8–11]. Although smoking has been shown to worsen diabetes-related complications, usually through recognized pernicious effects on circulation, the frequency of smoking among those with diabetes and the general population is comparable [12, 13]. Previous studies have also shown that smoking is associated with poor metabolic control [14, 15] and through this mechanism may play a part in accelerating the development of diabetic complications. However, most of these studies have not utilized longitudinal measurements of both glycemic control and smoking history in a large cohort of subjects with type 1 diabetes. Additionally, conflicting results have been reported on the risks associated with smoking on both nephropathy and retinopathy [11, 16–18].

In the present study, we assess the acute and long-term associations of smoking on glycemic control and diabetes-related complications using data from a well-characterized multicenter cohort of subjects with type 1 diabetes enrolled in the DCCT. Furthermore, we evaluate the extent to which glycemic control mediates the longitudinal association between smoking and complications.

## Materials and methods

### Study sample

The DCCT study has been described previously [19]. Briefly, the prospective, multicenter, randomized controlled clinical trial was designed to investigate whether intensive therapy, aimed at achieving glycemic control as close to the non-diabetic range as safely possible, would prevent the development and/or progression of diabetes-related complications [1]. Between 1983 and 1989, 1441 subjects with type 1 diabetes were enrolled in the study. Participants were 13 to 39 (mean 27) years old and were free of any advanced diabetes-related complications and other significant medical problems. Approximately one-half of the DCCT cohort (*n* = 711) was randomized to intensive therapy with a goal of maintaining blood glucose levels within a near-normal non-diabetic range. The remainder (*n* = 730) of the subjects were assigned to conventional therapy with a goal of clinical well-being and freedom from symptoms related to hyperglycemia or hypoglycemia. Two groups with varying levels of complications were recruited: the primary prevention cohort (*n* = 726) with 1–5 years diabetes duration, no retinopathy, and a urine albumin excretion rate (AER)<40 mg/24 hrs, and the secondary intervention cohort (*n* = 715) with 1–15 years of diabetes duration, mild to moderate non-proliferative diabetic retinopathy, and an AER≤200 mg/24 hrs.

Throughout the duration of the trial, 97% of subjects remained on their assigned treatment [20]. Deviations were primarily due to protocol-mandated changes (from conventional to intensive) in preparation for and during pregnancy. At the end of the DCCT, after an average of 6.5 years (range 3–9) of follow-up, 1422 participants (99% of the original cohort) completed a closeout visit (11 died and 8 lost to follow-up).

### DCCT evaluations and major outcomes

Subjects were evaluated quarterly. Each clinical visit included a detailed history and physical examination as well as assessment of glycemic control, measured by hemoglobin A1c (HbA1c). A central laboratory used high-performance liquid chromatography to assay all blood samples for HbA1c [21]. Other biochemical measurements performed locally and evaluated centrally included triglycerides, cholesterol levels, albumin excretion rate (AER), creatinine clearance, and serum creatinine.

Retinopathy was assessed by 7-field stereoscopic fundus photography and defined as a sustained progression at two consecutive 6-month visits of at least 3-steps in the Early Treatment Diabetic Retinopathy Study (ETDRS) score, relative to the level at baseline. Retinal photographs were taken every six months by certified DCCT photographers and centrally graded according to the procedures outlined in the ETDRS [22]. Nephropathy was assessed annually using 4-hour timed urine collections and evaluated centrally. Nephropathy was defined as microalbuminuria or worse based on a urinary AER≥40 mg/24 hrs.

### Smoking measurements

Data on smoking behaviors were collected annually during the DCCT by self-report. Subjects were asked whether they had ever smoked cigarettes in the past 12 months. If they were currently smoking, the average daily number of cigarettes was recorded. At baseline, information was also collected on the age at which participants became daily smokers, the number of years since first quitting smoking, the number of combined years that they did not smoke since first starting, and the average daily number of cigarettes while smoking.

In order to describe the ever-changing smoking behaviors throughout the course of the study, three categorical smoking status measurements were constructed as time-dependent covariates with values that were updated at each annual visit. The first measurement characterized subjects as never, former, or current smokers. At baseline, current smokers were defined as subjects who currently smoked or quit less than 3 months prior to baseline, former smokers as subjects who previously smoked but quit 3+ months prior to baseline, and never smokers as subjects who had never smoked prior to study entry. At each subsequent annual visit, never smokers could remain never smokers or become current or former smokers. Current and former smokers could only transition between the current and former smoking status categories.

The second definition of smoking status combined current and former smokers with lifetime pack-years to create a time-dependent variable that captured not only current smoking status but also lifetime intensity and duration. Current and former smokers were dichotomized as having smoked more or less than 10 pack-years over their lifetime, up to the date of their annual visit. Since all of the participants who entered the study as adolescents (*n* = 195) remained in the less than 10 pack-years category as current or former smokers, the analysis of smoking status and pack years could only be reliably conducted among adults. The third definition collapsed all current and former smokers into one category to summarize never smokers vs. ever smokers.

### Statistical analysis

Baseline demographic and medical characteristics were compared between never, former, and current smokers using the contingency chi-square test for categorical variables or the Kruskal-Wallis test for quantitative variables. Treatment group differences in smoking status were assessed using the contingency chi-square test. Generalized estimating equations (GEE) were used to test for DCCT treatment group differences in the odds of smoking (current vs. former and ever vs. never) over the duration of the study.

Generalized linear mixed models (GLMM) were used to test differences between-smoking groups in the annual HbA1c values over the study period. The repeated annual HbA1c measures were regressed on each of the three smoking status variables separately. Smoking status was included as a time-dependent covariate and all three models were minimally adjusted for time (DCCT study year) and treatment group. Each model was further adjusted for a set of baseline covariates that differed by smoking status and the interaction between smoking status and DCCT treatment group assignment was assessed.

Nelson-Aaalen estimates of the hazard rate for each one-year interval were estimated by the proportion of events (retinopathy or nephropathy) among those at risk at each DCCT study year. Separate Cox proportional hazards regression models were used to examine the effect of time-dependent smoking status on time to retinopathy and time to nephropathy after minimally adjusting for DCCT treatment group. In addition, the interaction between smoking status and DCCT treatment group assignment was evaluated. Seventy-four participants who entered the DCCT with an AER≥40 mg/24 hrs were excluded from the nephropathy models.

Mediation analyses were conducted to explore HbA1c as a mechanism that underlies the relationship between smoking and complications. Specifically, we sought to identify whether the association of smoking and complications is explained by the difference in HbA1c between smokers and non-smokers. It was hypothesized that after controlling for HbA1c, any previously significant effect of smoking on complications would either be diluted or no longer significant. Three steps were used to assess mediation of the relationship between smoking and complications by HbA1c. As described above, GLMM’s were used to examine the relationship between smoking and HbA1c (Step 2) and Cox proportional hazards regression models were used to examine the relationship between smoking and complications with and without the inclusion of the annual updated mean HbA1c as a time-dependent covariate (Steps 1 and 3, respectively). The percent of the total effect between smoking and complications explained by HbA1c was calculated as the percentage reduction in the beta estimate for smoking in Step 1 versus Step 3. Each model was further adjusted for a defined set of baseline covariates as well as for baseline retinopathy level and AER, and the percent of the total effect, explained by HbA1c as well as the additional baseline covariates, was reevaluated. All statistical analyses were performed using SAS version 9.3 (SAS Institute, Cary, NC).

## Results

Table 1 presents the demographic and medical characteristics of the DCCT cohort by baseline smoking status. At the start of the study, the mean difference in age was 5.2 years between former vs. never smokers and 3.9 years between current vs. never smokers (*P<0*.*01*). Twenty percent of never smokers were adolescents; the majority had also never been married (53%) and had some or no college education (61%). Additionally, only 16% of never smokers were current drinkers compared with 29% of former smokers and 34% of current smokers (*P<0*.*01*).

**Table 1:** Demographic and medical characteristics of participants in the diabetes control and complications trial by baseline smoking status.

	Never Smoker (*n* = 926)			Former Smoker (*n* = 221)			Current Smoker (*n* = 294)			*P* Value^a^
	No.	%	Mean (SD)	No.	%	Mean (SD)	No.	%	Mean (SD)	
**Study Design Parameters**										
Intensive treatment group	457	49.4		111	50.2		143	48.6		0.94
Primary prevention cohort	464	50.1		120	54.3		142	48.3		0.39
**Demographic Characteristics**										
Age, years			25.2 (7.3)			30.4 (5.5)			29.1 (5.9)	<0.0001
Adults	743	80.2		219	99.1		284	96.6		<0.0001
Males	481	51.9		115	52.0		165	56.1		0.44
Marital status										
Never married	489	52.8		59	26.7		101	34.4		<0.0001
Married or remarried	402	43.4		146	66.1		158	53.7		
Separated, divorced, widowed	35	3.8		16	7.2		35	11.9		
Occupation										
Professional or technical	297	32.1		85	38.6		68	23.9		<0.0001
Manager, official, proprietor, craftsman	101	10.9		34	15.5		73	25.6		
Student	317	34.3		17	7.7		36	12.6		
Other	209	22.6		84	38.2		108	37.9		
Education										
Graduate school	105	11.3		15	6.8		23	7.8		<0.0001
College graduate	253	27.3		80	36.2		54	18.4		
Some college	309	33.4		84	38.0		126	42.9		
Less than college	259	28.0		42	19.0		91	31.0		
Current drinker	144	15.6		65	29.4		101	34.4		<0.0001
Body mass index, kg/m^2^			23.3 (2.9)			23.8 (2.8)			23.3 (2.7)	0.072
Weight, kg			68.5 (12.2)			71.1 (11.9)			69.0 (11.4)	0.031
Level of exercise										
Strenuous	129	13.9		30	13.6		41	14.0		0.53
Hard	112	12.1		18	8.1		29	9.9		
Moderate	508	54.9		134	60.6		174	59.2		
Sedentary	177	19.1		39	17.7		50	17.0		
Total quality of life^b^			84.5 (14.4)			84.5 (15.5)			87.0 (15.5)	0.16
SCL-90R depression, T-score^c^			49.5 (10.1)			51.2 (8.6)			53.0 (11.0)	<0.0001
SCL-90R global severity index, T-score^c^			48.7 (10.0)			50.4 (8.8)			52.8 (12.3)	<0.0001
**Medical Characteristics**										
Diabetes duration, months			68.8 (49.7)			63.8 (49.9)			66.9 (50.6)	0.22
Cholesterol										
Total, mg/dL			173.7 (32.4)			178.1 (34.9)			183.5 (33.4)	0.0001
mmol/L			4.49 (0.84)			4.61 (0.90)			4.75 (0.86)	
HDL, mg/dL			50.8 (12.4)			52.4 (12.5)			48.4 (11.6)	0.0012
mmol/L			1.31 (0.32)			1.36 (0.32)			1.25 (0.30)	
LDL, mg/dL			107.5 (28.2)			110.0 (30.3)			116.4 (30.0)	<0.0001
mmol/L			2.78 (0.73)			2.84 (0.78)			3.01 (0.78)	
Triglycerides, mg/dL			77.6 (44.0)			79.0 (41.5)			94.6 (58.8)	<0.0001
mmol/mol			0.88 (0.50)			0.89 (0.47)			1.07 (0.66)	
Systolic blood pressure, mm Hg			114.5 (11.5)			112.6 (11.8)			113.9 (11.7)	0.038
Diastolic blood pressure, mm Hg			73.0 (8.8)			71.7 (9.1)			71.8 (8.9)	0.036
HbA1c, %^d^			9.0 (1.6)			8.9 (1.5)			9.4 (1.6)	<0.0001
mmol/mol			74.9 (17.9)			74.2 (16.8)			79.1 (17.2)	
AER, mg/24 hrs			16.0 (20.1)			13.3 (11.9)			17.6 (18.3)	0.0014
AER <30	821	88.7		208	94.1		255	86.7		0.022
AER 30–300	105	11.3		13	5.9		39	13.3		
Retinopathy level^e^										
No retinopathy in both eyes	464	50.1		120	54.3		142	48.3		0.55
Very mild NPDR in one or both eyes	296	32.0		62	28.1		91	31.0		
Mild NPDR in one or both eyes	96	10.4		21	9.5		29	9.9		
Moderate NPDR in one or both eye	70	7.6		18	8.1		32	10.9		

Abbreviations: AER, Albumin excretion rate; HbA1c, hemoglobin A1c; HDL, high-density lipoprotein; LDL, low-density lipoprotein; NPDR, Non-proliferative diabetic retinopathy; SCL-90R, Symptom Checklist-90-Revised; SD, standard deviation.

^a^ The *P* value evaluates the difference between all three categories of smoking using the contingency chi-square test for categorical variables or the Kruskal-Wallis test for quantitative variables.

^b^ 0 indicates the lowest quality of life score and 100 the highest quality of life score.

^c^ Psychiatric symptoms were assessed using the Psychiatric Symptom Checklist 90-R (SCL-90), a widely used and well-validated measure that provides an assessment of psychiatric symptoms and generates a total score on the global severity index and subscales, including depression. SCL-90 scores are converted to standard T-scores (ranging from 30–80) by referring to the appropriate population-based norm tables. T-scores have a mean of 50, std of 10, and a normal range of 40–60. A possible mental disorder is defined as a global severity index T-score ≥63.

^d^ The DCCT baseline HbA1c is the HbA1c value during the eligibility screening.

^e^ Retinopathy severity levels are defined by the final version of the ETDRS scale [22].

There were no differences in DCCT treatment group, cohort assignment, or duration of diabetes between current, former, and never smokers at baseline. Current smokers had higher levels of total cholesterol (mean difference 9.8 mg/dl, *P<0*.*01*), LDL cholesterol (8.9 mg/dl, *P<0*.*01*), and triglycerides (17.0 mg/dl, *P<0*.*01*), as well as lower levels of HDL cholesterol (-2.4 mg/dl, *P<0*.*01*) than never smokers. Current smokers at baseline had worse metabolic control compared with both former and never smokers (9.4% current, 8.9% former, and 9.0% never, *P<0*.*01*). Weight and blood pressure were marginally significant, favoring never smokers (*P<0*.*05*). AER levels were highest among current smokers, with 13% of the group ranging from 30 to 300 mg/24 hrs. All significant findings remained statistically significant when only adults were considered.

There were no significant differences between the DCCT treatment groups for any of the three smoking status variables (Table 2). At the start of the study, 294 (20%) of the 1,441 randomized participants were current smokers and 221 (15%) were former smokers. One hundred subjects who were never smokers at baseline now reported smoking during the trial, increasing the frequency of ever smokers (current plus former) from 515 (35%) at baseline to 615 (43%) by the end of follow-up. Although the proportion of current smokers was similar at baseline and at the end of follow-up (20% and 22%, respectively), the percentage of former smokers increased from 15% to 21% at study end.

**Table 2:** Smoking status by DCCT treatment group at baseline and at the end of an average of 6.5 years of follow-up.

	Baseline						End of follow-up					
		Overall	Intensive		Conventional		*P* Value^a^	Overall	Intensive		Conventional		*P* Value^a^
	No.	No.	%		No.	%	No.	No.	%	No.	%	
**Smoking status**												
	**(*n* = 1441)**	**(*n* = 711)**		**(*n* = 730)**			**(*n* = 1441)**	**(*n* = 711)**		**(*n* = 730)**		
Never smoker	926	457	64.3	469	64.3	0.94	826	413	58.1	413	56.6	0.81
Former smoker	221	111	15.6	110	15.1	303	145	20.4	158	21.6	
Current smoker	294	143	20.1	151	20.7	312	153	21.5	159	21.8	
Never smoker	926	457	64.3	469	64.3	0.99	826	413	58.1	413	56.6	0.56
Ever smoker	515	254	35.7	261	35.8	615	298	41.9	317	43.4	
*Among adolescents only*	**(*n* = 195)**	**(*n* = 92)**		**(*n* = 103)**			**(*n* = 195)**	**(*n* = 92)**		**(*n* = 103)**		
Never smoker	183	84	91.3	99	96.1	0.22	119	53	57.6	66	64.1	0.60
Former <10 pack-years	2	2	2.2	0	0.0	23	11	12.0	12	11.7	
Former ≥10 pack-years	0	0	0.0	0	0.0	0	0	0.0	0	0.0	
Current <10 pack-years	10	6	6.5	4	3.9	53	28	30.4	25	24.3	
Current ≥10 pack-years	0	0	0.0	0	0.0	0	0	0.0	0	0.0	
*Among adults only*	**(*n* = 1246)**	**(*n* = 619)**		**(*n* = 627)**			**(*n* = 1246)**	**(*n* = 619)**		**(*n* = 627)**		
Never smoker	743	373	60.3	370	59.0	0.97	707	360	58.2	347	55.3	0.45
Former <10 pack-years	150	74	12.0	76	12.1	195	87	14.1	108	17.2	
Former ≥10 pack-years	69	35	5.7	34	5.4	85	47	7.6	38	6.1	
Current <10 pack-years	156	77	12.4	79	12.6	95	46	7.4	49	7.8	
Current ≥10 pack-years	128	60	9.7	68	10.9	164	79	12.8	85	13.6	

Abbreviation: DCCT, Diabetes Control and Complications Trial.

^a^ The *P* value evaluates the treatment group differences using the contingency chi-square test.

Throughout the course of the DCCT, all of the participants who entered the study as adolescent smokers remained in the less than 10 pack-years category as current or former smokers (Table 2). Among those who entered the study as adult smokers, the majority of current smokers had accumulated less than 10 pack-years at baseline (*n* = 156) and more than 10 pack-years by the end of the study (*n* = 164). Therefore, the subsequent analyses that utilize the stratified smoking status variable have been restricted to participants who entered the study as adults.

Over the course of the study, the average proportion of both current smokers and ever smokers was slightly lower in the intensive group compared with the conventional group (21.1% vs. 21.5% current; 39.5% vs. 43.1% ever), however these differences were not statistically significant (current smokers odds ratio (OR) = 0.98, 95% confidence interval (CI): 0.03, 32.62; ever smokers OR = 0.86, 95% CI: 0.23, 3.24) (S1 Table).

### Smoking and HbA1c

**Fig 1:**
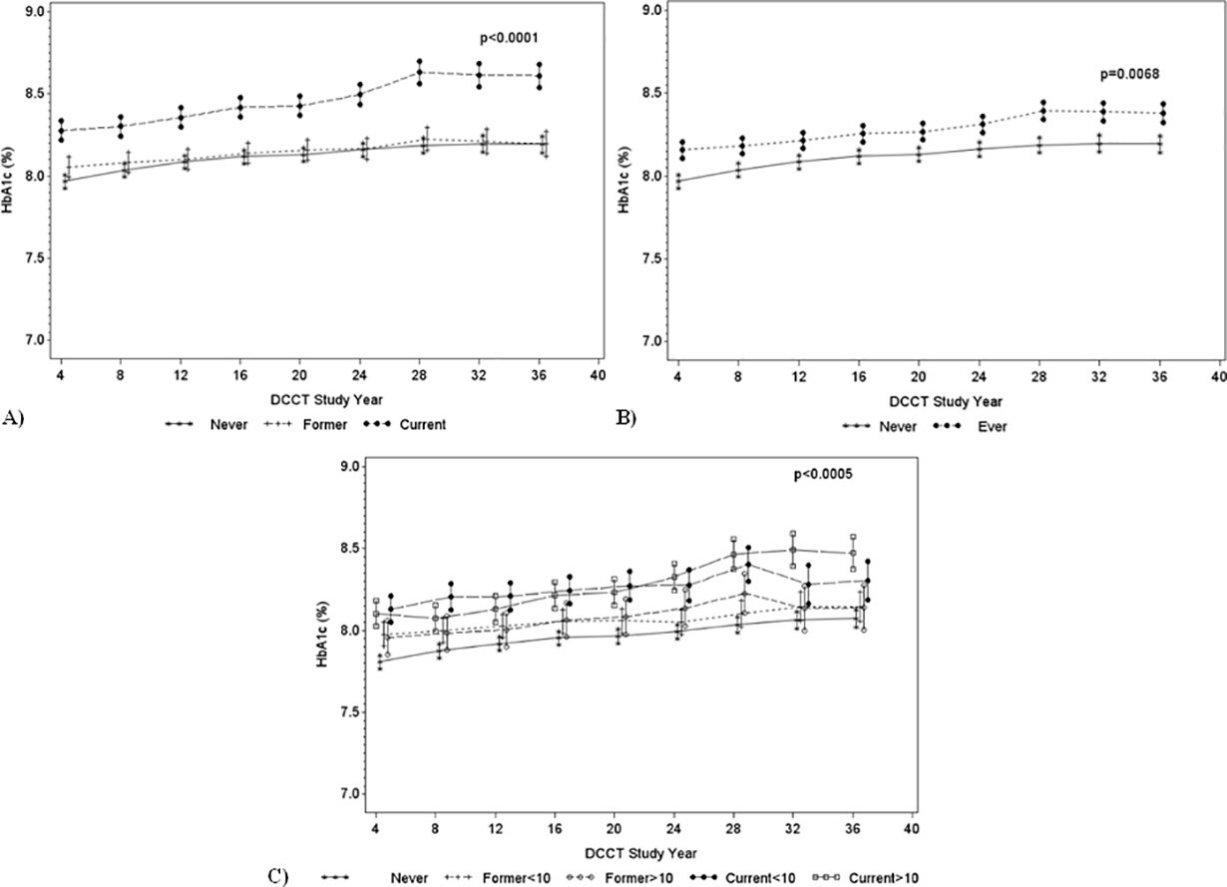
Mean HbA1c at each DCCT follow-up year by concurrent smoking status. (A) Never (stars), former (pluses), and current (black circles) smokers. (B) Never (stars) and ever (black circles) smokers. (C) never (stars), former<10 pack-years (pluses), former≥10 pack-years (white circles), current<10 pack-years (black circles), and current≥10 pack-years (white squares) smokers. Data are least squares means and standard errors obtained from three separate generalized linear mixed models presented in Table 3. Each model was minimally adjusted for time (DCCT study year), treatment group, an interaction between treatment group and time, and an interaction between smoking and time. Subjects may switch from one smoking category to another depending on their current status at each visit. (C) was restricted to 1,246 participants who entered the DCCT study as adults.

Mean levels of HbA1c averaged across all of the repeated measures were significantly different by smoking status (Fig 1 and Table 3), with current smokers having the highest mean HbA1c levels (average difference of 0.34% from never smokers, *P<0*.*01*; and 0.31% from former smokers, *P<0*.*01*). The mean HbA1c levels for former smokers were similar to those of never smokers. After collapsing current and former smokers into one category, the mean HbA1c levels were still significantly higher for ever smokers compared with never smokers (0.16%, *P<0*.*01*). Among the 1,246 participants who entered the study as adults, the mean HbA1c levels were highest for current smokers with more than 10 pack-years (average difference from never smokers 0.31%, *P<0*.*01*; and from former smokers with less than 10 pack-years 0.22%, *P<0*.*05*). Current smokers with less than 10 pack-years also had significantly higher mean HbA1c levels than never smokers (0.29%, *P<0*.*01*). There were no significant interactions between smoking status and treatment group.

**Table 3:** Associations of time-dependent smoking exposures with longitudinal HbA1c values in the diabetes control and complications trial.

	Model 1^a^^,^^b^		Model 2^a^^,^^c^			Model 1^a^^,^^b^		Model 2^a^^,^^c^	
	LS Means	*P* Value	LS Means	*P* Value	Significant Differences in LS Means	Difference	*P* Value	Difference	*P* Value
**Smoking status**									
Never smoker	8.12 (0.04)	<0.0001	8.14 (0.04)	0.0055	Current vs. Former	0.31 (0.08)	0.0001	0.15 (0.06)	0.023
Former smoker	8.15 (0.06)		8.17 (0.05)		Current vs. Never	0.34 (0.07)	<0.0001	0.18 (0.06)	0.0015
Current smoker	8.46 (0.06)		8.31 (0.05)						
Never smoker	8.12 (0.04)	0.0068	8.14 (0.04)	0.042	Ever vs. Never	0.16 (0.06)	0.0068	0.10 (0.05)	0.042
Ever smoker	8.28 (0.05)		8.24 (0.04)						
*Among adults only*									
Never smoker	7.97 (0.04)	0.0005	8.03 (0.04)	0.62	Current <10 vs. Never	0.29 (0.09)	0.0010	0.09 (0.07)	0.24
Former <10 pack-years	8.06 (0.07)		8.05 (0.06)		Current ≥10 vs. Never	0.31 (0.09)	0.0003	0.08 (0.08)	0.26
Former ≥10 pack-years	8.08 (0.10)		8.01 (0.08)		Current ≥10 vs. Former <10	0.22 (0.10)	0.035	0.07 (0.09)	0.44
Current <10 pack-years	8.26 (0.08)		8.12 (0.07)						
Current ≥10 pack-years	8.28 (0.08)		8.11 (0.07)						

Abbreviations: HbA1c, hemoglobin A1c; LS, least-squares; SD, standard deviation.

^a^ Data are from separate generalized linear mixed models regressing longitudinal HbA1c on smoking status. Smoking status was entered into each model as a time-dependent covariate. In each model, time (DCCT study year) was a significant main effect indicating that the mean HbA1c levels increased in each of the smoking categories over DCCT follow-up. There were no significant interactions between smoking and treatment group. The *P* value evaluates the overall significance of the fixed effects as well as the pair-wise differences in the least squares means.

^b^ Minimally adjusted for time (DCCT study year), treatment group, an interaction between treatment group and time, and an interaction between smoking and time.

^c^ Fully adjusted to also include age, gender, diabetes duration, education, drinking status, weight, total cholesterol, triglycerides, systolic and diastolic blood pressure, and baseline HbA1c.

After further adjustment for baseline covariates, the smoking effect on HbA1c was reduced with the difference in mean HbA1c between current and never smokers decreasing from 0.34% to 0.18%, but still remaining statistically significant (*P<0*.*01*). Ever smokers also continued to have significantly higher mean HbA1c levels (difference = 0.10%) compared with never smokers (*P<0*.*05*). There were no longer any significant differences in smoking status stratified by pack-years.

### Smoking and complications

**Fig 2:**
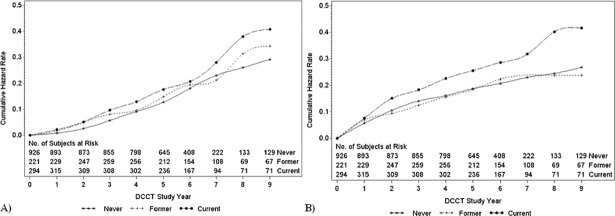
**Cumulative hazard rate of persistent 3-step change in ETDRS score relative to baseline (A) or nephropathy defined as an AER≥40 (B) during the DCCT by concurrent smoking status.** Never (stars), former (pluses), and current (black circles) smokers. Subjects may switch from one smoking category to another depending on their current status at each visit.

Over an average of 6.5 years of follow-up, 271 subjects developed retinopathy and 276 nephropathy (Fig 2 and Table 4). Current smokers had a 43% increased risk of retinopathy compared with never smokers (HR = 1.43 95% CI 1.08–1.89) and a 36% increased risk of nephropathy (HR = 1.36 95% CI 1.03–1.80). There were no significant differences between former and never smokers. After collapsing all current and former smokers into one category, ever smokers were at a slightly higher risk of retinopathy than never smokers (HR = 1.26 95% CI 1.00–1.61). Among adults, current smokers with more than 10 pack-years by the current visit were at the highest risk of nephropathy (HR = 1.59 95% CI 1.07–2.37), however there were no significant differences in the risk of retinopathy. Furthermore, there were no significant interactions between smoking status and treatment group.

**Table 4:** Mediation of HbA1c in the relationship between time-dependent smoking status and the risk of retinopathy and nephropathy.

		Model 1^c^		Model 2^c^^,^^d^		% Mediated^f^	Model 3^c^^,^^e^		% Mediated^f^
	No. of Events^b^	HR	95% CI	HR	95% CI		HR	95% CI	
**Retinopathy**	271								
Never smoker	144	1.00		1.00			1.00		
Former smoker	54	1.07	0.77, 1.49	1.19	0.85, 1.66	—	1.16	0.81, 1.64	—
Current smoker	73	1.43	1.08, 1.89	1.18	0.88, 1.57	54	1.09	0.80, 1.48	78
Never smoker	144	1.00		1.00			1.00		
Ever smoker	127	1.26	1.00, 1.61	1.18	0.93, 1.51	29	1.11	0.85, 1.46	56
**Nephropathy**^**a**^	276								
Never smoker	153	1.00		1.00			1.00		
Former smoker	47	0.80	0.56, 1.14	0.82	0.57, 1.17	—	0.99	0.68, 1.44	—
Current smoker	76	1.36	1.03, 1.80	1.21	0.91, 1.60	39	1.26	0.93, 1.72	22
Never smoker	153	1.00		1.00			1.00		
Ever smoker	123	1.10	0.86, 1.40	1.03	0.81, 1.32	—	1.15	0.88, 1.51	**—**
*Among adults only*									
Never smoker	104	1.00		1.00			1.00		
Former <10 pack-years	38	1.24	0.83, 1.85	1.22	0.81, 1.83	—	1.15	0.76, 1.75	—
Former ≥10 pack-years	7	0.55	0.24, 1.26	0.55	0.24, 1.26	—	0.82	0.34, 1.95	—
Current <10 pack-years	19	1.28	0.81, 2.01	1.16	0.74, 1.84	—	0.99	0.61, 1.60	—
Current ≥10 pack-years	34	1.59	1.07, 2.37	1.42	0.95, 2.12	25%	1.54	0.97, 2.44	7%

Abbreviations: AER, albumin excretion rate; CI, confidence interval; HR, hazard ratio.

^a^ Seventy-four participants who entered the DCCT with an AER greater than or equal to 40 mg/24 hrs were excluded from the nephropathy models.

^b^ Number of events while in each state.

^c^ Data are from separate Cox proportional hazards models regressing either retinopathy or nephropathy on smoking status, minimally adjusted for DCCT treatment group. In each model, the interaction between smoking status and treatment group was not significant. Smoking status was entered into each model as a time-dependent covariate.

^d^ Simultaneously adjusting for *both* HbA1c and smoking status as time-dependent covariates.

^e^ Simultaneously adjusting for *both* HbA1c and smoking status as time-dependent covariates. Fully adjusted to also include age, gender, diabetes duration, education, drinking status, weight, total cholesterol, triglycerides, systolic and diastolic blood pressure, and baseline HbA1c. The retinopathy models also adjusted for baseline retinopathy level while the nephropathy models adjusted for baseline AER.

^f^ Percent of the total effect between smoking and complications that is mediated by HbA1c or by HbA1c in combination with other baseline covariates, calculated as the percentage reduction in the beta estimate for smoking status in Model 1 versus Model 2 or 3.

### Mediation

As described above, current smoking status was significantly associated with HbA1c and both retinopathy and nephropathy. After including HbA1c as a time-dependent covariate in the Cox proportional hazards regression models, the increased risks of retinopathy and nephropathy among current smokers were no longer statistically significant (Table 4). For current vs. never smokers, the hazard ratio decreased from 1.43 to 1.18 in the retinopathy model and from 1.36 to 1.21 in the nephropathy model, representing 54% and 39% (respectively) of the total effect between smoking and complications explained by HbA1c alone. After further adjusting each model for other baseline covariates, the hazard ratio for smoking was further attenuated in the retinopathy model, representing 78% of the total effect explained by HbA1c and the baseline covariate effects. The percent of the total effect explained by all of the covariate adjustments in the nephropathy model remained below 50%.

The marginally significant relationship between ever smokers and retinopathy decreased from 1.26 to 1.18 after adjustment for HbA1c (29% of the total effect) and further decreased to 1.11 after including all baseline covariates in the model (56% of the total effect). Finally, among adults, the increased risk of nephropathy for current smokers with more than 10 pack-years, decreased from 1.59 to 1.42 after adjustment for HbA1c, representing 25% of the total effect explained by HbA1c alone. As observed with current vs. never smokers, the percent of the total effect explained by all of the covariate adjustments in the nephropathy model remained below 50%.

## Discussion

The DCCT provides an opportunity to examine whether smoking behaviors measured over time correlate with glycemic control and the risk of microvascular complications in type 1 diabetes. Over an average of 6.5 years of follow-up, current smokers consistently exhibited worse glycemic control compared to both former and never smokers. Current smokers were also at a higher risk of retinopathy and nephropathy. These effects were attenuated after adjustment for HbA1c, suggesting that the relationship between smoking and complications is predominately mediated by differences in HbA1c between smoking categories.

The relationship between smoking and both HbA1c and complication status has been previously studied in both type 1 and type 2 diabetes populations [14, 23, 24]. These studies have shown that smoking increases blood glucose, impairs glucose tolerance and is an independent risk factor for type 2 diabetes [14, 25–28]. The DCCT has also clearly established that elevated blood glucose levels increase the risk of microvascular complications [29, 30]. However, the association between smoking and the risk of complications has been inconsistent and few studies have utilized longitudinal measures of smoking and also examined the mediating effects of HbA1c. The current analysis is the first to present the associations in a large sample of subjects with type 1 diabetes using longitudinal measures of smoking, HbA1c, and complication status.

Around the time that the DCCT ended, two studies reported significant mean absolute differences in HbA1c between smokers and nonsmokers. The EURODIAB Complications Study, a large prevalence survey of 3,250 subjects with type 1 diabetes, reported a 0.6% mean difference in males (6.9% vs. 6.3%) and a 0.3% difference in females (6.9% vs. 6.6%) [31] while Chase et al reported an overall mean difference of 0.6% (7.9% vs. 7.3%) in a population-based study of 359 subjects with type 1 diabetes (mean age 20) [8]. More recently, Gerber et al also observed a mean difference of 0.6% (12.1% vs. 11.5%) between smokers and nonsmokers in a prospective study of 763 subjects with type 1 diabetes (mean age 36) over an average follow-up of 5.7 years [32]. The present analysis demonstrated significant differences in mean HbA1c, but of smaller magnitude (absolute difference 0.34%; relative difference 4%). The mean HbA1c values in the EURODIAB and Gerber studies were similar to those observed during the DCCT, specifically in the INT treatment group, while the mean HbA1c values in the Chase study were much higher due to the fact that the participants were younger and most were not utilizing intensive diabetes regimens. Similar to the current analysis, both the EURODIAB and Chase studies demonstrated that ex-smokers could achieve equivalent levels of glycemic control compared to nonsmokers.

In the present analyses, current smokers had a 43% increased risk of retinopathy and a 36% increased risk of nephropathy compared with never smokers. Furthermore, current smokers with more than 10 pack-years were at the highest risk of developing nephropathy (HR = 1.59). In previous studies, the effects of smoking on the risk of microvascular complications have been inconsistent. Although Chase, Gerber, and the EUROBDIAB prevalence study all demonstrated an association between smoking status and nephropathy [8, 31, 32], others have not [33–35]. The reported associations between smoking and retinopathy have mostly been negative [16, 17, 36], however, both the EURODIAB prevalence study and the Chase study did show modest significant differences between smokers and nonsmokers. It is possible that inconsistent findings in the relationship between smoking and complications might be due to selective mortality. Subjects who smoked may have died prior to experiencing a microvascular event, thereby biasing the results towards the null. However, in the DCCT, 99% of the original cohort completed the study.

The significant associations between smoking and complications were attenuated by the effects of HbA1c. When comparing the risk of complications between current smokers and never smokers, the percent of the total effect that was mediated by HbA1c was 54% for retinopathy and 39% for nephropathy. In previous studies, the attenuation of the effects of smoking on the risk of complications after adjusting for HbA1c, has been inconsistent. The EURODIAB prevalence study showed that adjustment for current or long-term HbA1c accounted for most of the differences between smokers and nonsmokers, however the associations remained statistically significant [31]. The Scott and Chase studies also found that the associations between smoking and nephropathy decreased but remained significant after further adjustment for HbA1c [8, 9].

The current results demonstrate that former smokers do not significantly differ from never smokers in both the mean level of HbA1c and the risk of complications. This suggests that former smokers can achieve similar glycemic control to those who never smoked and consequently also decrease their risk of microvascular complications. Other studies have also shown that the risks for former smokers parallel those of nonsmokers [8, 31]. It is therefore hypothesized that the negative effects of smoking on HbA1c and the risk of complications may not persist after quitting smoking, or that smoking does not have a negative memory effect on glycemia and complication risk.

The generalizability of the results of this study may be limited due to the strict control of study procedures in a clinical trial environment. Subjects in the DCCT received more medical care than is generally offered in a clinical setting. Additionally, children under 13 years of age, adults over 40 years of age, and subjects with a history of frequent hypoglycemia or severe complications were excluded at study enrollment.

## Conclusions

In conclusion, this study confirms that smoking is associated with poor glycemic control and an increased risk of microvascular complications in type 1 diabetes. Elevated blood glucose levels, caused by the negative effects of smoking, account for most of the significant association between smoking and complications. Individuals with type 1 diabetes who smoke, have significantly worse metabolic control and are therefore at a greater risk of developing complications. This work highlights the importance of smoking as a major modifiable risk factor in the development of microvascular complications in type 1 diabetes and reinforces the importance of smoking cessation. As detailed throughout, former smokers can achieve similar glycemic control to never smokers and reduce their risk of complications. The results of this study should be used to encourage individuals with type 1 diabetes to avoid smoking or to quit as soon as possible.

## Supporting information

S1 Table Prevalence of smoking status by DCCT treatment group at baseline and at each study year. (DOCX)
