# Supplementary material for: Mediation of the association of smoking and microvascular complications by glycemic control in type 1 diabetes
Source: PLoS One. 2019 Jan 7;14(1):e0210367. doi: 10.1371/journal.pone.0210367 (PMC6322792; doi:10.1371/journal.pone.0210367)
Supplement: S1 Table — (DOCX) [file pone.0210367.s001.docx]

**S1 Table. Prevalence of Smoking Status by DCCT Treatment Group at Baseline and at Each Study Year.**

|  | **DCCT**  **Baseline** | **Year 1** | **Year 2** | **Year 3** | **Year**  **4** | **Year 5** | **Year 6** | **Year 7** | **Year 8** | **Year 9** | **Longitudinal Model^a^** | | |
| --- | --- | --- | --- | --- | --- | --- | --- | --- | --- | --- | --- | --- | --- |
|  |  |  |  |  |  |  |  |  |  |  | **Average**  **Proportion** | **OR** | **95% CI** |
|  |  |  |  |  |  |  |  |  |  |  |  |  |  |
| **Overall, *n*** | 1,441 | 1,437 | 1,429 | 1,422 | 1,357 | 1,094 | 729 | 425 | 273 | 268 |  |  |  |
| Intensive | 711 | 709 | 703 | 697 | 672 | 549 | 378 | 215 | 142 | 140 |  |  |  |
| Conventional | 730 | 728 | 726 | 725 | 685 | 545 | 351 | 210 | 131 | 128 |  |  |  |
|  |  |  |  |  |  |  |  |  |  |  |  |  |  |
|  |  |  |  |  |  |  |  |  |  |  |  |  |  |
| **Prevalence of current smokers, %** | | | | | | | | | | |  |  |  |
| Intensive | 20.1 | 21.0 | 21.9 | 21.4 | 22.2 | 20.8 | 23.8 | 24.7 | 27.5 | 27.9 | 21.1 | 0.98 | 0.03, 32.62 |
| Conventional | 20.7 | 22.8 | 21.4 | 21.9 | 22.5 | 22.4 | 21.9 | 20.0 | 24.4 | 25.0 | 21.5 |  |  |
|  |  |  |  |  |  |  |  |  |  |  |  |  |  |
|  |  |  |  |  |  |  |  |  |  |  |  |  |  |
| **Prevalence of ever smokers, %** | | | | | | | | | | |  |  |  |
| Intensive | 35.7 | 37.4 | 38.8 | 39.2 | 41.1 | 39.9 | 44.4 | 49.8 | 50.0 | 50.7 | 39.5 | 0.86 | 0.23, 3.24 |
| Conventional | 35.8 | 38.3 | 39.0 | 40.6 | 41.3 | 42.0 | 43.6 | 45.7 | 52.7 | 52.3 | 43.1 |  |  |
|  | | | | | | | | | | |  |  |  |

Abbreviations: CI, confidence interval; DCCT, Diabetes Control and Complications Trial; OR, odds ratio.

^a^ The longitudinal model presents data from two separate repeated measures logistic regression models comparing the odds of smoking between subjects in the intensive and conventional treatment groups, after adjusting for time (DCCT study year). In both models, the interaction between treatment group and time was not significant. In model 2, time (DCCT study year) was a significant positive main effect indicating that the proportion of ever smokers increased in the two treatment groups over the course of the trial. However, since the interaction between treatment group and DCCT study year was not significant, the rate at which smoking increased was similar among the intensive and conventional subjects. The average proportion of smokers by treatment group and the odds ratios (95% confidence intervals) are presented.
